# Simple yet effective modifications to the operation of the Sediment Isolation Microplastic unit to avoid polyvinyl chloride (PVC) contamination

**DOI:** 10.1016/j.mex.2019.11.007

**Published:** 2019-11-09

**Authors:** Holly Nel, Stefan Krause, Gregory H. Sambrook Smith, Iseult Lynch

**Affiliations:** School of Geography, Earth and Environmental Sciences, University of Birmingham, Edgbaston, Birmingham, B15 2TT, UK

**Keywords:** Sediment Isolation Microplastic unit, Extraction, Density separation, Negative blank, Positive blank, Percent recovery

## Abstract

Effective microplastic extraction from sediment and soil samples requires a density separation step, with the ability to remove >80 % of plastic particles without introducing substantial contamination. Additional benefits such as affordability and simplicity allow microplastic campaigns on limited budgets the ability to achieve high extraction efficacies. Coppock et al. (2017) designed the Sediment Microplastic Isolation (SMI) unit with these criteria in mind, warning that long-term use may lead to polyvinyl chloride (PVC) contamination. As part of the method validation work for a large-scale international project, collecting samples from more than 100 rivers globally, a pilot study of extraction efficiency and contamination potential of an SMI unit was performed. PVC contamination occurred during the extraction of 20 samples, with indicative grey shavings found in both negative controls and field samples. The original protocol was modified and artificially spiked sediments (positive blanks) were run to test extraction efficacy. The modification, requiring the PVC ball valve to remain open throughout the extraction. This modification eliminated contamination caused by wear and tear of the ball valve, while still maintaining recovery rates >80 %.

Three points describing the change not the original:

• The PVC ball valve is open while sample is agitated with a magnetic stirrer.
• The PVC ball valve remains open while the solution is decanted.
• The upper chamber is unscrewed and rinsed; recovering particles attached to the inner walls that would be lost using other filtration approaches.

## Graphical abstract

**Figure:**
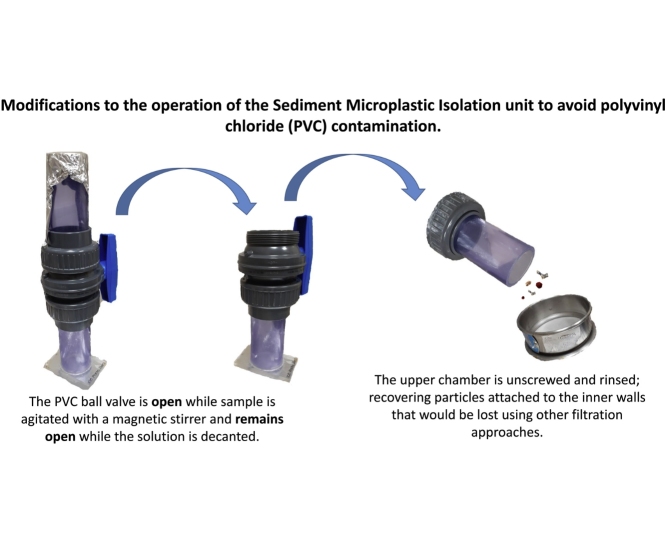

**Specification Table**

**Table:** 

Subject Area:	*Environmental Science*
More specific subject area:	*Microplastic environmental contamination*
Method name:	*Sediment Microplastic Isolation unit;* [1]
Name and reference of original method:	*Coppock, R.L., Cole, M., Lindeque, P.K., Queiros, A.M. and Galloway, T.S. (2017) A small-scale, portable method for extracting microplastics from marine sediments. Environmental Pollution 230, 829-837.*
Resource availability:	*N/A*

## Method details

### Background

**Fig. 1:**
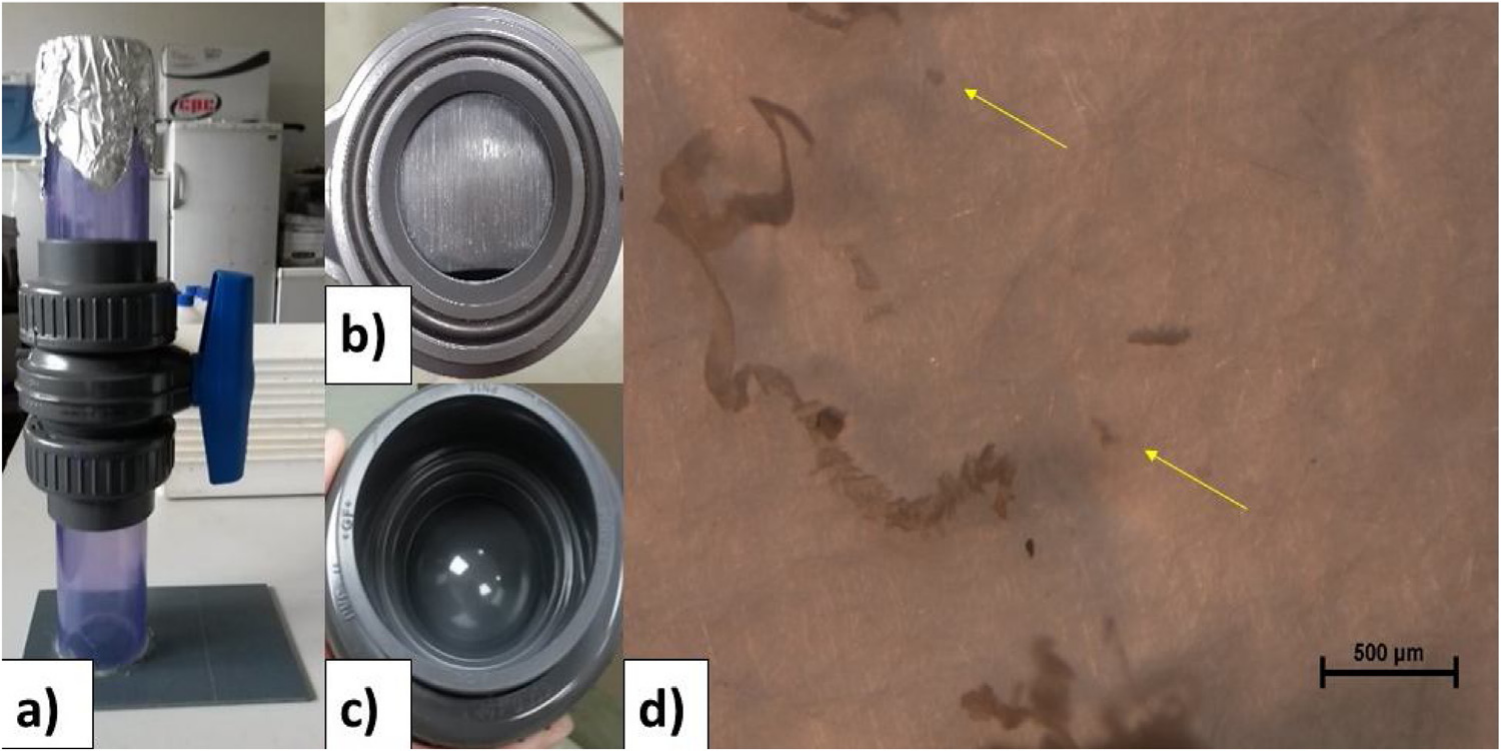
Sediment Microplastic Isolation unit constructed at the University of Birmingham (Image a); Ball valve after being used to process 20 sediment and blank samples showing striations (Image b); New ball valve showing no striations (Image c); Polyvinyl chloride shavings found in blanks. Yellow arrows show particles <200 μm (Image d). Note that similar shavings where found in field samples, suggesting that the smaller particles are likely also introduced into the field samples confounding any quantification.

As part of method validation work for future microplastic sampling campaigns, a pilot study of extraction efficiency and contamination potential of a Sediment Microplastic Isolation (SMI) unit designed by Coppock et al. [1] was performed using estuarine sediments collected in the United Kingdom (UK). Estuarine sediments were collected using clean glass jars (225 mL), previously rinsed with distilled water, by the Clean Seas Odyssey team (https://www.cleanseasodyssey.org/) from 18^th^ June to 30^th^ August 2018 in partnership with the University of Birmingham. A sub-set (N = 20) was processed using an SMI unit (Fig. 1; Image a) with zinc chloride (ZnCl_2_; 1.5 g cm^−3^) solution as per the original Coppock et al. [1] protocol.

In summary, sediment samples arrived at the University of Birmingham where they were immediately oven dried at 50 °C for 72 h. To avoid airborne contamination the oven was cleaned thoroughly before use and glass jars were covered with clean tinfoil. Once constant weight was reached a sub-sample, between 30 and 50 g, was taken for extraction procedures. Sediment was weighed and stored in clean stainless steel dishes, using a balance (accurate to the nearest 0.1 g). The SMI unit was filled with 700 mL ZnCl_2_ solution and primed before use. This required the ball valve to be opened and closed 3–5 times in a process which lubricates the ball valve, while simultaneously filling the air pockets with ZnCl_2_. Thereafter 30–50 g of dry sediment was introduced into the chamber along with a magnetic stirring bead. The SMI unit was then placed on a magnetic stirrer for 10 min at a stirring level between 5 and 8 rpm. Sediment may remain on the surface due to the viscosity of the ZnCl_2_ solution. This was combatted by stirring the mixture with a glass rod, which was then cleaned with filtered ZnCl_2_. The unit was covered with clean tinfoil when in use to avoid the introduction of airborne contamination. After the sample was sufficiently homogenised it was left to settle for 10 min. This duration was extended to 2 h in certain cases; for example when samples contained a high proportion of fine particles. Ultimately, it is important to allow time for majority of the denser particles to settle out, therefore resulting in cleaner samples. The ball valve was then closed trapping the supernatant in the upper chamber. The supernatant was poured over a stainless steel 63 μm mesh, taking particular care to rinse the upper chamber thoroughly with deionized water. Matter remaining on the mesh was then transferred to a 20 mL glass vial and stored for further analyses. Simultaneously, negative blanks were run using the same procedure, excluding the sediment. Striations were evidenced on the ball valve after 20 extractions (Fig. 1; Image b) that were not originally documented (Fig. 1; Image c) suggesting a risk of polyvinyl chloride (PVC) contamination of the samples. This was confirmed by analysing blank samples, which contained grey fragments/shavings (Fig. 1; Image d) further characterised as being PVC using a multihyphenated thermogravimetric analysis-Fourier transformed infrared spectroscopy-gas chromatography-mass spectrometry (TGA-FTIR-GC-MS) set-up. Sediment samples were analysed and shown to contain shavings similar in colour and shape to those recorded in the blank samples. PVC shavings >200 μm were counted and ranged between 0 and 11 per sample (negative blank controls and field sediment samples, with the numbers increasing in samples processed towards the end of the sub-set). Above 200 μm the shavings were clearly indicative of contamination, however, small particles (Image D – yellow arrows) could not be definitively confirmed as being contamination or not, suggesting that simply removing PVC contamination from total microplastic counts would not be feasible. Contamination occurring within the processing time of 20 samples suggests that long-term use of this unit may lead to overestimation of PVC polymers in field samples. It must be noted that this phenomenon occurred even after the SMI unit had been primed, which was suggested by Coppock et al. [1] to avoid this from occurring. As a result of this contamination, a modification to the operation of the SMI unit was proposed which is presented herein.

### Method validation

Ultimately, the unit design has remained the same. In the modified protocol the ball valve is not utilised, therefore there is no need to open and close the ball valve before introducing the sediment or to close the ball valve once the denser sediment particles have settled out. This prevents wear and tear of the ball valve, which results in sample contamination shown in Fig. 1(b). The supernatant is subsequently decanted similar to traditional methods, however, the SMI unit design allows the upper chamber to be removed and rinsed thereby avoiding the loss of particles attached to the edge of the equipment walls. This cannot be done in traditional methods, which utilise a glass beaker, thus providing a motivation to retain the SMI unit even without using the ball valve.

**Fig. 2:**
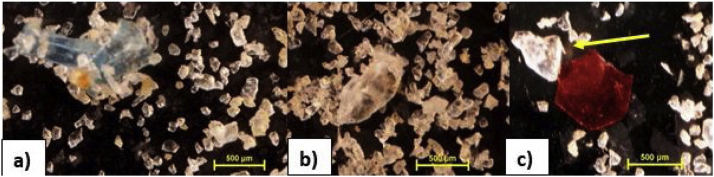
Microplastics of various polymer compositions (Polypropylene – Image a; Polyethylene terephthalate – Image b and c (Red particle); clear polyethylene (Image c – yellow arrow)) recovered during positive blank method validation tests.

Positive blanks (sediment samples spiked with plastic particles) were run eight times using the modified protocol. These positive blanks contained 10 Polyethylene terephthalate (PET) fragments and either 10 Polyethylene (PE) or 10 Polypropylene (PP) fragments (Fig. 2), resulting in 20 particles spiked into 30 g of clean sediment and homogenised gently. The microplastics were extracted using the SMI unit and protocol described above, with the ball valve open. Supernatant was concentrated on a 63 μm stainless steel mesh and washed into new petri dishes. After 24 h of oven drying at 50 °C five researchers independently analysed the positive blanks. The particle recovery rates were calculated by dividing the numbers of recovered particles by the number added initially.

There appears to be differences in observer counts (Table 1), which could be attributed to individual bias and experience. Four out of the five observers had between 1 and 6 months experience, while the observer with the highest percent recovery (Observer C) had more than five years experience of working with microplastics. An analysis of variance (ANOVA), however, showed no significant difference between the individual observer recovery rates (high-density polymer and low-density polymer: p > 0.05). It may be suggested that equipment validation is subject to some degree of visual bias which may vary depending on experience level, particle size and colour, although this needs further testing. Overall, however, both high-density and low-density polymers were recovered using this protocol, and taking the most experienced observers results averaged at 92 ± 4 (SE) and 88 ± 5 (SE) % respectively (Table 1).

**Table 1:** Percent (%) recovery rates for high- and low- density polymers by five independent observers using the modified Sediment Microplastic Isolation unit protocol.

	High-density polymer (PET)			Low-density polymer (PE or PP)		
Observer	Percent recovery	Standard error (SE)	Range	Percent recovery	SE	Range
A	87	7	60–100	83	6	60–100
B	86	7	60–100	79	5	60–100
C	92	4	80–100	88	5	70–100
D	83	9	50–100	81	6	50–100
E	89	5	70–100	85	6	60–100
Overall Average	87	3	50–100	83	2	50–100

The suggested modification was also validated by analysing negative blanks (sediment free ZnCl_2_ solution) throughout the extraction of 150+ samples. The 15 blank samples, performed after every 10 sample extractions or once a day, did not evidence any indicative shavings, suggesting that PVC contamination had been successfully prevented. This was done while maintaining a high recovery rate of between 81 (inexperienced user) and 92 % (experienced user) (Table 1).

## Conclusion

In conclusion, the proposed modification utilises the original SMI unit and thus simple design and inexpensive cost. However, without continuously closing and opening the ball valve, contamination caused by the resulting friction is substantially reduced. The protocol described here may be used for polymer extraction from a range of sediment types (i.e. marine, estuarine and river sediments, soils); however, adjustment to settling times may be required for finer grained environmental matrices. Additionally, before a field sampling project begins positive controls should be run using sediment typical of the environment under investigation to verify its suitability and record recovery rates alongside results to improve reproducibility and comparability between studies. Inclusion of periodic negative blanks to confirm no contamination from the SMI unit is also recommended. To date, the authors are not aware of any published field studies which have utilised the original SMI operational protocol and are not suggesting such studies are substantially contaminated. However, this simple modification to prevent potential PVC contamination eliminates the risk of substantial contamination due to wear and tear of the ball without compromising on recovery efficiency.

## Additional information

Sediment contaminated with microplastics, defined as primary (produced as) or secondary (breakdown from larger) plastic particles of less than 5 mm in diameter, have been recorded in marine [2], freshwater [3] and estuarine environments [4] as well as the lower atmosphere [5]. The recent increase in microplastic research has seen a surge in the development of methodological techniques aimed at enhancing our ability to accurately identify and quantify this contaminant in a range of environmental matrices. Identification of microplastics can be challenging due to the wide range and complexity of different synthetic polymers found in the environment as well as the abundance of other entities of similar sizes and densities making their identification and extraction from sediments a challenge. Thus, effective approaches for the separation of microplastics from the other constituents of sediment are vital. For example, extracting microplastics from bulk sediment samples requires a density separation step [6], which conventionally favoured polymers such as polyethylene (PE), polypropylene (PP) and polystyrene that have a lower density than the solution used for their extraction [7]. Low recovery rates also result from particles adhering to equipment walls and subsequent reintroduction to the sediment fraction [6].

Method validations that yield sufficiently high extraction efficacy from intentionally spiked test samples are imperative in order to increase confidence that the chosen method can effectively recover all microplastics present in field samples where concentrations are unknown. To overcome and improve polymer recovery rates, a number of alternative approaches have been suggested namely, centrifugation (97 %; [8]), elutriation (98–100 %; [9]), the Munich Particle Sediment Separator (96–100 %; [10]; 13–39 % and 97. %; [11]) and the Sediment Microplastic Isolation unit (96 %; [1]).

In this technical note, we discuss the Sediment Microplastic Isolation (SMI) unit designed by Coppock et al. [1], which provides a semi-portable, inexpensive solution for polymer extraction from sediments with reported recovery rates between 92 and 98 % for both low- and high-density polymers. Affordability is attained by constructing the unit from polyvinyl chloride (PVC), a polymer that has been recorded in microplastic polluted field samples [12,13]. A PVC ball valve is used to compartmentalise the sediment fraction from the supernatant allowing the upper section to be rinsed multiple times [1]. Although, this unit provides a promising technique for microplastic extraction there is a risk of equipment contamination with PVC during prolonged use. Coppock et al. [1] recognised this downside stating that long-term use had not been tested in the method validation. The introduction of microplastics from external sources (i.e. equipment contamination, lab contamination, liquid contamination, air contamination, clothing contamination) needs to be minimised as it could seriously compromise the accuracy of microplastic estimates at a sample site.

## Declaration of Competing Interest

The authors declare that they have no known competing financial interests or personal relationships that could have appeared to influence the work reported in this paper.
